# The older people, omega-3, and cognitive health (EPOCH) trial design and methodology: A randomised, double-blind, controlled trial investigating the effect of long-chain omega-3 fatty acids on cognitive ageing and wellbeing in cognitively healthy older adults

**DOI:** 10.1186/1475-2891-10-117

**Published:** 2011-10-20

**Authors:** Vanessa Danthiir, Nicholas R Burns, Ted Nettelbeck, Carlene Wilson, Gary Wittert

**Affiliations:** 1 Preventative Health Research Flagship, Commonwealth Scientific and Industrial Research Organisation - Food and Nutritional Sciences, Adelaide, South Australia, Australia; 2 School of Psychology, University of Adelaide, Adelaide, South Australia, Australia; 3 School of Medicine, University of Adelaide, Adelaide, South Australia, Australia; 4 School of Medicine, Flinders University, Adelaide, South Australia

## Abstract

**Background:**

Some studies have suggested an association between omega-3 long-chain polyunsaturated fatty acids (*n*-3 LC PUFAs) and better cognitive outcomes in older adults. To date, only two randomised, controlled trials have assessed the effect of *n*-3 LC PUFA supplementation on cognitive function in older cognitively healthy populations. Of these trials only one found a benefit, in the subgroup carrying the ApoE-ε4 allele. The benefits of *n*-3 LC PUFA supplementation on cognitive function in older normal populations thus still remain unclear. The main objective of the current study was to provide a comprehensive assessment of the potential of *n*-3 LC PUFAs to slow cognitive decline in normal elderly people, and included ApoE-ε4 allele carriage as a potential moderating factor. The detailed methodology of the trial is reported herein.

**Methods:**

The study was a parallel, 18-month, randomised, double-blind, placebo-controlled intervention with assessment at baseline and repeated 6-monthly. Participants (N = 391, 53.7% female) aged 65-90 years, English-speaking and with normal cognitive function, were recruited from metropolitan Adelaide, South Australia. Participants in the intervention arm received capsules containing fish-oil at a daily dosage of 1720 mg of docosahexaenoic acid and 600 mg of eicosapentaenoic acid while the placebo arm received the equivalent amount of olive oil in their capsules. The primary outcome is rate of change in cognitive performance, as measured by latent variables for the cognitive constructs (encompassing Reasoning, Working Memory, Short-term Memory, Retrieval Fluency, Inhibition, Simple and Choice-Reaction Time, Perceptual Speed, Odd-man-out Reaction Time, Speed of Memory Scanning, and Psychomotor Speed) and assessed by latent growth curve modeling. Secondary outcomes are change in the Mini-mental State Examination, functional capacity and well-being (including health status, depression, mood, and self-report cognitive functioning), blood pressure, and biomarkers of *n*-3 LC PUFA status, glucose, lipid metabolism, inflammation, oxidative stress, and DNA damage.

**Trial registration:**

Australia and New Zealand Clinical Trials Register (ANZCTR): ACTRN12607000278437

## Background

Ageing is accompanied by what is normally considered to be inevitable cognitive decline, although the extent to which this occurs is highly variable, and strongly affected by various disease processes. Cognitive function is a major determinant of quality of life in older age and decline in cognitive functioning is a primary contributing factor to increasing dependency in the elderly. Due to the ageing profile of the population, the financial, social, and other burdens that this dependency places upon society are of increasing concern. Thus, finding ways to prevent or ameliorate age-related cognitive decline is a public health imperative, with potential benefits not just in terms of lessening aged care costs but also in the enhancement of well-being in a growing segment of society.

Omega-3 polyunsaturated fatty acids (*n*-3 PUFAs) are one diet-related factor suggested to influence cognitive decline during ageing. The *n*-3 long-chain (LC) PUFAs eicosapentaenoic acid (EPA) and docosahexaenoic acid (DHA) are crucial to brain development and normal brain functioning [1]. DHA is particularly important to brain functioning due to its influence on neural membrane properties, which modulate cell signalling [2]. DHA concentration in the brain decreases with age in humans [3] and rats [4]; this has been postulated to be consequential to the age-related deterioration in central nervous system functions [3]. Evidence from animal studies supports this; animals fed a low *n*-3 PUFA diet show cognitive deficits [4,5] that are ameliorated by DHA supplementation [6,7] and DHA supplementation improves memory performance in aged mice [8].

### Cross-sectional and prospective studies

Oily fish are the major dietary source of the *n*-3 LC PUFAs, EPA and DHA. A consistent finding across studies is that higher intake of fish is related to less cognitive decline [9-12] and incidence of dementia [10,13,14] in prospective studies, and associated with better cognitive performance in non-clinical samples [11,15-17], in cross-sectional analyses. However, variable associations have been found between dietary intake levels of *n*-3 LC PUFAs and cognitive outcomes; only a handful of the aforementioned studies that also examined relationships between cognitive outcomes and dietary intake levels of *n*-3 LC PUFAs have found significant positive relationships [9,14,15].

Stronger evidence of a positive relationship between cognitive outcomes in older age and levels of *n*-3 LC PUFAs has been found in studies assessing plasma or erythrocyte levels of *n*-3 PUFAs. In these studies, DHA and EPA levels, either individually or in combination, have been associated with better cognitive function in normal older adults in cross-sectional analyses [18,19], and in prospective studies, with better cognitive outcomes over time [19] or reduced risk of cognitive decline [20], and lower risk of developing dementia [21]; in contrast, Laurin et al [22] reported a negative relationship between cognition and biomarkers of *n*-3 LC PUFAs.

### Randomised controlled trials (RCTs)

There have been six published double-blind RCTs of *n*-3 LC PUFAs on cognitive functioning in the elderly within both clinical and non-clinical populations [23-28]. Only two studies have been conducted with cognitively healthy populations, screened for dementia at baseline, and no overall effect of supplementation was found. The "Older People and *n*-3 Long-chain polyunsaturated fatty acids" study [24] supplemented 867 cognitively healthy participants aged 70-79 years, with either 200 mg of EPA and 500 mg of DHA, or olive oil, daily for 24 months. After adjustment for confounders, there was no significant difference between the groups in performance on the California Verbal Learning Test, the main cognitive outcome measure, or any secondary cognitive outcomes (a global cognitive score, and domain scores on memory, processing speed, executive function, and delayed recall). In a 26-week RCT of fish oil on cognitive performance in 302 cognitively healthy adults aged 65+ years [27], no main effect of supplementation was seen for any of the cognitive domains (attention, sensorimotor speed, memory, and executive function), assessed by 5 tests. However, subgroup analysis revealed that carriers of the Apolipoprotein E (ApoE) ε4 allele, in both the low (226 mg EPA, 176 mg DHA) and high dose (1093 mg EPA, 847 mg DHA) fish oil groups, showed an improvement after 26 weeks compared with the placebo on the Digit-Span Forward task, their marker of attention.

In three of the studies with cognitively impaired (including Alzheimer's disease (AD)) samples, the interventions had a beneficial effect on cognitive outcomes but only in participants with mild cognitive dysfunction [23,25,28]. This finding was not replicated in a large RCT conducted over 18 months of 402 participants with mild to moderate AD. However, in this study in ApoE-ε4 non-carriers only, there was a positive effect on two cognitive outcome measures [26].

### Apolipoprotein E-ε4 allele

The presence or absence of the Apolipoprotein E-ε4 allele appears to be a potentially important modifier of relationships between *n*-3 LC PUFAs and older-age cognitive outcomes but there are conflicting findings regarding the direction of this interaction. Epidemiological studies that have reported a differential relationship between cognitive outcomes and measures of *n*-3 LC PUFAs for carriers versus non-carriers of the ApoE-ε4 allele have found a beneficial relationship present only in non-carriers [19,29]. However, the two RCTs that have included examination of the effect of ApoE-ε4 allele carriage in sub-analyses have reported a beneficial effect of supplementation dependent on ApoE-ε4 allele status, but differ on which group benefits from supplementation [26,27]. Nonetheless, ApoE polymorphisms have been found to influence lipid responses to fish oil supplementation [30] and in a small group of males, those without ApoE-ε4 showed a greater increase in DHA and EPA after fish-oil supplementation [31]. Further, possession of the ApoE-ε4 allele is associated with worse cognitive performance in old age [32] and greater risk of cognitive decline and dementia [33], hence, even in cognitively healthy samples screened for dementia, the genesis of any cognitive change may differ between carriers and non-carriers, providing another potential avenue for differential effects of supplementation on cognitive outcomes due to ApoE genotype.

These studies suggest that *n*-3 LC PUFA intake, in particular DHA, has a beneficial effect on cognition in older adults. However, the majority of evidence for the benefits of *n*-3 PUFA on cognitive functioning in adults is associational. Furthermore, work to date in this area has often not comprehensively assessed cognitive function or cognitive decline. Without *a priori *expectations of specific cognitive domains that will be affected, a comprehensive operationalisation of cognition is required in order to ascertain whether a null result is due to the absence of a relationship or effect, or the relevant cognitive domains were not assessed. Given the potential importance of ApoE-ε4 allele carriage as a determinant of *n*-3 LC PUFAs influence upon cognitive outcomes in older age, more randomised controlled trials are needed that incorporate consideration of its impact in order to determine whether *n*-3 LC PUFAs can influence age-related cognitive change in older adults.

## Methods/Design

The primary aim of the current trial was to comprehensively assess over 18-months the efficacy of a DHA-rich fish-oil supplement on slowing cognitive decline in non-demented older adults; the hypothesis was that, over 18 months, the *n*-3 LC PUFA supplementation group would show slower cognitive decline than the control group. The trial was set within the broader context of examining nutritional, health, and lifestyle factors associated with better cognitive and well-being outcomes in the elderly, both cross-sectionally and longitudinally.

Secondary aims were: 1) to explore relationships between measures of *n*-3 LC PUFA intake and measures of cognitive function at baseline; 2) to determine whether the effect of *n*-3 LC PUFA supplementation on cognitive decline is moderated by presence of the ApoE-ε4 allele, initial cognitive status, measures of dietary *n*-3 LC PUFA intake (including changes in *n*-3 LC PUFA status) or any nutritional, health-related, or psychological factors associated with cognitive outcomes in the elderly; 3) to examine the effect of the intervention on measures of functional capacity and well-being; and 4) examine relationships between measures of *n*-3 LC PUFA intake and functional capacity and well-being at baseline.

This paper details the methodology of the study, including the design, assessment battery, and research protocols used.

### Study design

The trial was a parallel, 18-month, randomised, double-blind, placebo-controlled trial with repeated measures every 6 months, totalling 4 measurement points. Because the primary hypothesis related to change in a continual developmental process, multiple repeated measures enable determination of the rate of change on the measures, allowing assessment of an intervention effect on a trajectory, rather than effects at a specific time point. Decline of the cognitive abilities vulnerable to ageing effects begins by the early 30s so although participants were screened for cognitive impairment, all were expected to experience cognitive decline to some extent; for instance, meta-analyses of cross-sectional data suggest that processing speed declines, on average, by approximately 20% at age 40 and by 40-60% at age 80 [34]. In terms of supplementation duration, studies have detected significant cognitive changes due to *n*-3 PUFA supplementation after 3-4 months [35,36]. However, these studies focused on clinical populations; change in normal populations would not be expected to be as rapid, or of the same magnitude and the longer duration allowed for the multiple testing points required to best test the hypothesis and conferred greater power to detect an effect.

The trial was co-ordinated from CSIRO Food and Nutritional Sciences, Adelaide, Australia. The protocol was explained to participants and written informed consent was obtained prior to study commencement. All experimental procedures were approved by the Human Research Ethics Committee of CSIRO and in compliance with the Helsinki declaration. The study follows Good Clinical Research Practice. Participants were encouraged to inform the trial research officer of any adverse events; these were recorded by the officer. Adverse effects suspected as being related to supplement use were reported to the trial clinical doctor for follow-up. Participants attended the centre every 3 months; every 6 months, to undergo their assessments, and 3-months after each assessment to obtain additional capsules and be followed-up. At these visits, adverse events were recorded, along with any changes in medication, supplement use, or general health.

### Participant recruitment

#### Sampling and criteria

Participants were recruited from metropolitan Adelaide, South Australia and surrounding areas via agencies and organisations for older citizens, public advertisements, and media releases. Information sheets explaining the study and asking for volunteers, were circulated in response to expressions of interest. To be eligible for inclusion, participants were required to be 65-90 years of age at screening, fluent in English, and agree to not commence their own *n*-3 fish-oil (or algal) supplementation throughout the duration of the study. Participants were excluded from study entry if they were taking *n*-3 fish-oil (or algal) supplements, had experienced any condition where cognitive impairment may be a consequence (e.g., head injury, other brain trauma, transient ischemic attacks, stroke, coronary artery bypass surgery, open heart surgery, degenerative neurological disease, history of alcohol/drug abuse), were taking doses of medications known to interfere with cognition, had a significant medical condition, were diagnosed with an intellectual disability, current major clinical depression, diabetes, dementia, or received a score less than 22/27 on a telephone-administered version [37] of the Mini-mental State Examination (MMSE) (equivalent to < 24/30 on the standard MMSE, the traditional cut-off for possible dementia).

#### Screening and information sessions

Individuals who expressed interest in participating were sent a questionnaire to obtain demographic information, history of health events, and specific questions to determine eligibility. Participants deemed eligible, based on the questionnaire responses, were contacted by a trained research officer and, if consenting, were administered a version of the MMSE via telephone - this method of administration has been validated for the MMSE [37]. Participants scoring less than 22 correct out of a potential 27 points were excluded. Subsequently, approximately two to three months prior to baseline testing, participants attended an information session, during which the study protocol and requirements were explained to them, queries were answered, and practice trials and examples of the cognitive tasks were given. Participants were asked to maintain their usual diet over the course of the study. After written consent to participate was obtained, individual administration of the standard MMSE was undertaken, to corroborate the telephone screening and to serve as an outcome measure.

#### Randomisation, allocation, and blinding

Participants were given a unique study number on entry into the trial. An independent researcher prepared allocation to treatment. Age-stratified, permuted-block randomisation, with mixed block-sizes ranging from two to eight (size unknown to study investigators until unblinding), with 1:1 allocation was employed. Participants were placed into 5-year age strata, and randomisation for each stratum followed a computer generated randomisation schedule. The researchers, project staff, and participants remained blinded to treatment allocation until the trial was completed and the database locked. Capsule bottles were labelled with participants' unique study numbers by an external company. Active and placebo capsules (opaque brown, oblong, and softgel; Blackmores Pty Ltd, Australia) and containers were visually identical, the fish-oil was the low-odour type, and 1% fish-oil was added to the placebo oil to help maintain blinding in the event of, for example, accidental piercing of the capsules or an aftertaste. At the study-end, participants were asked to guess which study arm they were in, to assess adequacy of blinding.

### Dietary intervention

#### Dietary intervention schedule

The intervention was administered for 18 months, starting immediately after baseline assessment, after which participants received their first 3-month supply of capsule bottles. For the study duration, participants returned to the CSIRO clinic every 3 months, received their next 3-month supply of capsules, and returned all unused capsules. The trial research officers recorded any reported side-effects of the capsules.

#### Compliance

Compliance was monitored via a count of all unused supplements returned at three-monthly intervals, along with self-report calendars, mailed back on a monthly basis throughout the trial. If participants' compliance fell below 85% on the basis of their calendars, they were contacted by a research officer who noted the reasons. Compliance also will be assessed by examination of erythrocyte membrane *n-*3 LC PUFA status and changes in status, via a blood sample taken at every 6 month-assessment.

#### *n*-3 LC PUFA capsule

The supplement was chosen to be DHA-rich on the basis that evidence suggests DHA is more related to cognition in ageing, than is EPA. The dosage for the current trial followed that of the first reported *n*-3 LC PUFA placebo-controlled intervention study focusing on cognitive decline in the elderly (albeit in AD patients [25]) that found a positive effect; hence, the study dose was 1720 mg DHA and 600 mg EPA daily. Each capsule contained 430 mg DHA and 150 mg EPA (EPAX 1050 TG/N *n*-3 concentrate, EPAX, Norway); participants were instructed to have a total of four capsules a day, two in the morning and two in the evening. The total *n*-3 LC PUFAs in the daily supplement were equivalent to approximately 150 g of salmon. There are no known risks associated with *n*-3 LC PUFA consumption at this level. Evidence supports the effectiveness of *n*-3 fish-oil supplements as providers of *n*-3 LC PUFAs, with *n*-3 LC PUFAs from fish-oil supplements being incorporated into erythrocyte membranes to a similar extent as *n*-3 LC PUFAs from dietary fish intake [38].

#### Placebo capsule

The placebo arm received capsules, each containing 990 mg of olive oil (Oil Seed Products, New Zealand) and 10 mg of fish oil (equivalent to 1.8 mg EPA and 1.2 mg DHA; omega-3 18/12, Ocean Nutrition, Canada) and consumed a total of 4 capsules a day. Unlike other oils, olive oil is not rich in *n*-3 or *n*-6 fatty acids and should thus have minimal effect on the *n*-6:*n*-3 ratio. Olive oil is high in mono-unsaturated fatty acids (MUFAs), which have in some epidemiological studies been associated with lower cognitive decline [39], yet it is thought that it is the polyphenolic content of the oil that might bear this effect. The amount of MUFAs in the capsules was very minor compared to the dietary consumption of MUFAs in those studies that found a cognitively protective effect (i.e., mean daily MUFA intake = 42.1 g [39]). The 4 g of low-polyphenol olive oil per day was thus considered non-problematic. The minimal amount of fish oil was added to help mask any difference between the placebo and active capsules and was not processed under nitrogen, rendering it less potent than that used in the active formulation.

### Assessment Administration

After the group information session, participants were scheduled one visit to the laboratory for assessment, every 6 months, for 18 months. Self-report questionnaires (see following section on study instruments) were mailed out to participants up to four weeks prior to each assessment visit, with instructions to complete and return them between one to two weeks prior to their assessment. Missing data or incorrectly completed questionnaires were thus identified beforehand and addressed during their assessment sessions. All physical measurements and cognitive testing were conducted at the same time in the morning at each session.

For each assessment visit, participants attended the clinic after an overnight fast, and a blood sample and physical measurements (height (at baseline only), weight, and blood pressure) were undertaken by trained phlebotomists and staff. Participants were then given a standardised breakfast in the centre, prior to undertaking the cognitive test battery. At baseline and final assessment, blood was taken for measurement of the following. Carotenoids, folate, and vitamin B12, were assessed, all having been associated with cognitive functioning in the elderly; this enables examination of cross-sectional associations between these measures and outcomes, and if necessary in the intervention analyses, to control for any potential differences between groups at baseline or any changes in these measures that differ between groups (despite randomisation). Glucose, glycated haemoglobin (HbA1c), lipid concentrations (total cholesterol, high-density lipoprotein (HDL), low-density lipoprotein (LDL), and triglycerides), and homocysteine were also measured at baseline and final assessment, because of associations between abnormalities in these parameters of metabolism and cognitive dysfunction [30]. Inclusion of these measures allows us to control for possible baseline differences between groups and potentially assess whether any changes on these measures due to the intervention mediate changes on the main outcome variables. Finally, our blood-derived measures included a high sensitivity assay for C-reactive protein (CRP), an inflammatory marker, plasma malondialdehyde (MDA), a marker of oxidative stress, and telomere length, a marker of oxidative stress and DNA damage. Oxidative stress and inflammation have been associated with cognitive decline or impairment [40,41] and are two postulated mechanisms through which *n*-3 LC PUFAs may exert an effect on cognitive functioning [34,35]; thus, we will examine cross-sectional associations between these measures and our cognitive outcomes, be able to control for potential differences between groups at baseline, and assess whether any changes due to fish oil supplementation mediate any change on the primary outcomes. Apolipoprotein E (ApoE) genotyping was also conducted in order to examine any differential effect of treatment due to carriage of the ApoE-ε4 allele. Blood was taken at the second and third assessment points in order to assess erythrocyte membrane fatty acid composition at each time point. *n*-3 LC PUFA status will be used to assess compliance/uptake post-hoc and we will also assess whether changes in *n*-3 LC PUFA status are associated with any changes on our outcomes. EPA levels in erythrocytes reflect intake over the past month or two, change linearly with dosage (up to 9 g/day), and increase is evident after 3 days of supplementation; EPA and DHA are incorporated equally effectively [42]. Dietary data for current *n*-3 and *n*-6 fatty acid intake was gathered at all time-points, to assess stability of dietary LC PUFA intake over the trial, a food frequency questionnaire (FFQ) was administered as a measure of typical nutrient intake over the preceding one-year period at baseline and at Time 4, and historical seafood consumption was also assessed. Cross-sectional associations between these measures and outcomes will be investigated, and if there are differences between groups at baseline or changes in *n*-3 or overall dietary intake which differ significantly between the active and control groups, then we will control for any potential effects of this on outcomes. Table 1 summarises the data collected at each time point.

**Table 1:** Summary of measures assessed over the study duration.

Outcome measures and covariates	Time points (months)				
	
	~ -3	0	6	12	18
MMSE	x	x			x

Cognitive measures		x	x	x	x

Physical activity, functional capacity, and well-being measures		x	x	x	x

Height		x			

Blood pressure, weight		x	x	x	x

Erythrocyte membrane *n*-3 and *n*-6 fatty acid status		x	x	x	x

Serum folate and Vitamin B12, and plasma carotenoid levels		x			x

Lipids, glucose, glycated haemoglobin, homocysteine		x			x

Medications, supplement use, adverse events and health changes		x	x	x	x

ApoE genotype		x			

Inflammation (CRP), oxidative stress (MDA), and telomere length		x			x

Dietary data - general FFQ		x			x

Dietary data - *n*-3 consumption FFQ		x	x	x	x

Dietary data - historical seafood consumption FFQ			x		

Abbreviations: MMSE = Mini-mental state examination; *n*-3 = omega-3; *n*-6 = omega-6; FFQ = food frequency questionnaire; ApoE = Apolipoprotein E.

### Outcome measures

#### Primary outcome measures

Rate of change in cognitive performance, as measured by latent variables for the cognitive constructs detailed below.

#### Secondary outcome measures

Change in: MMSE; perceived health status, depressive symptoms, positive and negative affect, life satisfaction, self-reported cognitive functioning, and functional capacity, as assessed by the questionnaires below; blood pressure; biomarkers of glucose, glycated haemoglobin, triglycerides, total cholesterol, HDL, LDL, homocysteine, CRP, MDA, and telomere length.

### Cognitive test battery

No previous definitive evidence suggests which cognitive domains may be amenable to change via *n*-3 LC PUFAs, thus the test battery used in this study comprehensively assessed all relevant domains that might benefit from a nutritional intervention. The focus of the battery was on constructs that are sensitive to age-related changes and that are most strongly associated with functional status, functional decline, and dementia.

Most of the cognitive domains were assessed using tasks in which speed of response was the main outcome measure. The psychometric properties of speed tasks make them more sensitive to subtle cognitive changes and obviate the need for parallel forms for repeated assessment. These properties, together with evidence that processing speed is integral to higher-order processes such as reasoning [43], may be a key indicator of changes in neural efficiency [44] and functional outcomes [45], and is an important mediator of age-related cognitive changes [46], make speed tasks ideal as outcome measures to test the effect of a nutritional intervention on cognitive performance in older-age.

With the exception of two individual tasks, at least two measures of each cognitive construct were used to indicate a latent construct for statistical analyses, thereby reducing task-specific variance and increasing construct validity and the reliability of assessment. The cognitive constructs assessed were Reasoning, Working Memory, Short-term Memory, Retrieval Fluency, Inhibition, Simple and Choice-Reaction Time, Perceptual Speed, Odd-man-out Reaction Time, Speed of Memory Scanning, Psychomotor Speed, Inspection Time, and Knowledge. The latter two were measured by only one task each and the knowledge measure was included as a 'control' task in that the construct it assesses does not decline with age nor would it be expected to be modifiable by intervention. Excepting the tasks measured by speed of response, parallel versions of each task were presented at each assessment visit unless otherwise specified. For a detailed description of the test battery see Appendix 1.

All measures were completed in small groups of up to 7 people, in CSIRO's Cognitive Laboratory, and administered by 2 trained research officers. The testing sessions lasted approximately four hours and consisted of four sections of around 50 min duration, with a break of approximately 10 min between each section. During this break participants were provided with standard refreshments, including decaffeinated tea/coffee. The speed tasks were interspersed between the accuracy based tasks and to also help minimise fatigue, paper and pencil tasks were interspersed with computerised tasks, which were administered in an adjacent room. The order of tasks was fixed across participants and testing sessions. Instructions and practice items preceded each test, and questions were clarified before commencement.

### Participant demographics

At study commencement, a questionnaire ascertained the following demographic and health information: sex, date of birth, and occupation (prior main occupation if retired); diagnosed medical conditions and regularly taken medications; supplement use and duration; and, for women, the use of hormone replacement therapy. A smoking questionnaire assessed current smoking status, the number of cigarettes/cigars/pipes smoked per day, and smoking history, from which pack years was derived (number of pack years = (number of cigarettes smoked per day × number of years smoked)/20) to measure lifetime direct smoking exposure. Changes to smoking habits were captured at study end.

Information was updated regarding medical and health details during the first and then subsequent visits to the CSIRO clinic.

During the assessment sessions, questionnaires were administered to gather other relevant demographic information: highest level of education achieved, country of birth and if relevant, the age of arrival in Australia and years of speaking English, parents' birth country and occupations, perceived ethnicity, cultural determinants of family diet in childhood, familial history of diagnosed dementia, current income level (as per the Australian census, 2006), and relationship status. Frequency of computer use, handedness, and possible hearing and vision impairments (including colour blindness) were also recorded. Participants' level of engagement in day-to-day mental or cognitive activities was assessed by self-reported minutes per week doing challenging puzzles, self-directed learning, managing finances, or teaching/attending classes. At study end participants stated which experimental group they thought they were in, to assess adequacy of blinding.

### Physical activity, functional capacity, and well-being

To assess changes in functional capacity and well-being, and potentially control for any effect of changes in mood or physical activity on cognitive functioning, validated questionnaires assessing everyday functioning (The Late Life Function and Disability Instrument [47,48]), depression (Centre for Epidemiology Studies Depression Scale [49]), self-reported cognitive functioning (Cognitive Failures Questionnaire [50], Prospective and Retrospective Memory Questionnaire [51]), subjective well-being (mood: Positive and Negative Affect Scale-Extended [52]; life satisfaction: Diener's Satisfaction with Life Scale [53], the Personal Wellbeing Index [54]), health-related quality of life (SF-36 [55], the sleep scale from the Nottingham Health Profile [56]), and physical activity (The Yale Physical Activity Survey [57]) were compiled as a general health and wellbeing questionnaire, which participants completed for each assessment. See Appendix 2 for descriptions of the questionnaires and derived scores.

### Dietary Intake

The Victorian Cancer Council FFQ [58,59] was used to measure average daily dietary intake, and a Polyunsaturated Fatty Acid FFQ [60] was used to assess usual dietary intake of *n*-3 and *n*-6 PUFAs. The PUFA FFQ was administered in paper format and scored using the electronic version. Lifetime seafood intake was assessed via the Lifetime Diet Questionnaire [61].

### Anthropometry and blood pressure

Height was measured at baseline, using a wall-mounted stadiometer (SECA, Hamburg, Germany) and weight was measured at each assessment session using a calibrated digital precision scale (UC-321PBT; A&D Medical, Sydney, Australia). Body Mass Index was calculated as weight (kg)/height (m)^2 ^to examine as a potential covariate. Blood pressure was measured in the left arm and after the subject had been seated supine for 5 minutes, using an automated sphygmomanometer (Sure Signs V3; Philips Medical Systems, Andover, MA), and the average of three consecutive readings was used as the measured value for each assessment.

### Biochemical indicators

Blood was collected into Vacutainer tubes containing EDTA, cooled to 4°C and separated into plasma (for malondialdehyde and carotenoids analyses) and erythrocytes (for analysis of fatty acids) by centrifugation. Erythrocytes were washed twice with isotonic saline and reconstituted to the original volume in water to lyse the cells. For glucose analyses, plasma was separated from blood collected into Vacutainer tubes containing EDTA + NaF. Serum was separated from clotted blood for lipid analyses. Aliquots of plasma, serum and lysed erythrocytes were stored at -80°C until analysis.

Serum vitamin B12 and folate, plasma homocysteine, and HbA1c were measured at an accredited pathology laboratory (IMVS, Adelaide, Australia). Plasma glucose and serum total cholesterol, triglycerides, HDL, and CRP were measured on a Hitachi 902 clinical analyser using diagnostic kits (Roche Diagnostics, Australia). LDL cholesterol was calculated using the Friedewald Equation [62]. Plasma MDA was measured by a fluorescence high performance liquid chromatography (HPLC) method as detailed by Belobradjic et al [63].

Carotenoids were measured on a Shimadzu LC10 HPLC fitted with a refrigerated auto sampler and a SPD-MV10 Avp photodiode array detector with a class LC10 chromatography workstation. The chromatographic separations of the fat soluble vitamins and carotenoids were carried out on a Varian Microsorb-MV reverse phase C18 column, 4.6 mm ID × 250 mm length, 5 micron spherical particles [64].

The analysis of erythrocyte membrane fatty acids was based on a method reported by Ridges et al. [65], which uses a one-step extraction and transesterification procedure [66]. Solid phase extraction on florisil (Sigma-Aldrich Pty Ltd, Castle Hill, NSW Australia) was used to clean up the toluene extract containing the fatty acid methyl esters (FAME) after the transesterification procedure. Briefly, the toluene was dried under nitrogen, residue redissolved in hexane and loaded onto florisil. The FAME were eluted from the florisil with 10% ether in hexane, dried under nitrogen and re-dissolved in isooctane. An aliquot was injected onto a bonded-phase vitreous silica BPX70 column 30 m × 0.53 mm × 0.5 micron (SGE, Australia) in an Agilent 6890N gas chromatograph equipped with a cool on-column injector. Peak identification was based on a comparison of retention times with Supelco 37 component FAME mix 47885-U (Sigma-Aldrich Pty Ltd, Castle Hill, NSW Australia) and peak areas were measured using ChemStation software.

### Genomic Markers

ApoE genotyping was performed using Polymerase Chain Reaction (PCR) - Restriction Fragment Length Polymorphism method, as described by Hixson et al. [67]. Absolute telomere length was measured by determining the number of TTAGGG hexamer repeats using quantitative real-time PCR as described by O'Callaghan et al [68].

### Statistical Considerations

#### Analysis

Latent growth curve modelling (LGCM) will be the main analysis method used to assess the effect of the intervention. LGCM is an appropriate and rigorous methodology to employ for intervention studies that aim to alter a developmental trajectory and has greater power to detect an effect than models traditionally used in examining interventions, such as ANCOVA or repeated measures ANOVA [69].

Four equally-spaced testing time points were used in this study because, firstly, four time points are required to distinguish between different growth forms (e.g., linear, quadratic) and, secondly, four time points are estimated to give adequate power. The cognitive domains will be modelled as latent variables, providing the cognitive factors prove to be measuring the same construct across time, following the initial planned modelling of the tasks and cognitive factors on baseline data using confirmatory factor analysis (CFA). The main effect of the intervention on each cognitive domain will initially be assessed separately following Muthèn and Curran [69]. The main intervention effect can be tested by modelling the shape of the curve, for the time-points of a construct, for treatment and control groups separately. Both groups are constrained to the same normative growth trajectory, and the treatment slope added to the treatment group. Statistical significance of the mean of the treatment slope indicates a significant main treatment effect. Within this framework, further tests will also examine interaction effects, to determine whether the magnitude of the treatment effect varies as a function of initial cognitive status, and whether other factors, such as carriage of the ApoE-e4 allele, sex, or initial *n*-3 status, moderate the treatment effect. Similar analyses will be undertaken for well-being.

#### Power

Power was estimated based on a small effect size (Cohen's d) of .2, because there was no solid basis for an expected effect size, and followed power analysis based on LGCM of an artificial longitudinal treatment dataset [69]. This effect size equated to a difference in slope of .23 SD of the control group slope (set at 20% of initial status). Power here refers to the power to detect a difference between the treatment and control groups in terms of mean rate of decline and/or in the variance of the decline rate at an alpha level of .05. A total sample-size of 350 was required for power of .84. To detect an interaction effect of .40 (moderate size) between initial status and treatment slope in the treatment group, with a main effect of .2, required a sample-size ≌ 275. We aimed for an initial sample of 420, allowing for an attrition rate of approx. 17%, resulting in *N *= 350 at time 4, giving sufficient power to detect the primary effects.

### Data treatment of cognitive indicators

Treatment and analysis of reaction time data was conducted only on trials with correct responses. Unfeasibly long latencies on single trials and latencies less than 200 ms or greater than 3.5 intraindividual standard deviations above the individual's mean on a particular task were set to missing, and individuals' mean latencies then recomputed. Considering the above factors, if a participant had less than 60% of valid trial data on a particular task, their mean reaction time for the task was set to missing. Participants' scores were also set to missing on a particular speed task if their accuracy was less than 70% on that task. For the speed tasks and excepting the inhibition tasks' difference scores, the inverse of mean correct latencies were used, to normalise the distribution [70] and to express performance as a work rate. For all tasks (both speed and accuracy based), total or mean reaction time scores for data for tasks not completed, or not completed according to correct procedure, were considered missing.

### Modifications to cognitive test battery after baseline assessment

Confirmatory factor analyses were planned on the baseline data (N = 391) to assess the relationships between the tasks and constructs and, in particular, to ensure no empirical redundancy between any of the constructs. The cognitive test battery was potentially to be abbreviated for future assessments, on the basis of the outcomes.

**Figure 1:**
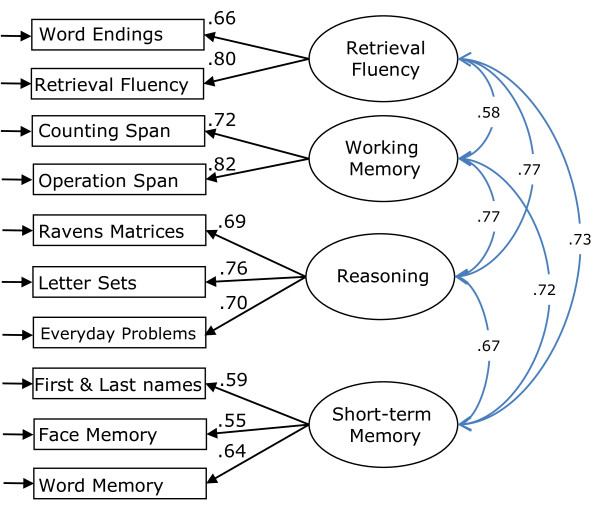
**Measurement Model for the tasks assessing level of cognitive performance**. Final measurement model of the baseline data for the accuracy-based tasks (χ^2 ^= 53.8, *df *= 29, *p *= .003; RMSEA = .047, 90%CI [.027, .066]; CFI = .979; AIC = 125.8). The Face Memory and Word Memory indicators represent the immediate recall/recognition versions. Correlations are represented by double-headed arrows.

CFA was performed separately on the baseline data for the tasks assessed by accuracy and speed of response, respectively, using full information maximum likelihood estimation in AMOS 7, to deal with the small amount of missing data. For the accuracy based tasks, latent variables were specified for Fluid Intelligence, Working Memory, Short-term Memory, Long-term Memory, and Retrieval Fluency, indicated by the respective tasks specified in Appendix 1 under each construct. The latent variable covariances were free to vary and the residual variances of the immediate and delayed versions of the two memory tasks were allowed to covary to account for their shared methods. All loadings were of at least moderate magnitude, significant, and in the expected direction and the model fit the data well (χ^2 ^= 66.42, *df *= 42, *p *= .010; Root Mean Square Error of Approximation (RMSEA) = .039, 90%CI [.019, .056]; Comparative Fit Index (CFI) = .984; Akaike Information Criterion (AIC) = 162.4); however, the covariance matrix among the factors was not positive definite because the correlation between Long-term Memory and Short-term Memory was near unity (r = .975). To examine whether both factors were necessary, a model was specified without Long-term Memory and with the delayed memory tasks loading on Short-term Memory (χ^2 ^= 71.5, *df *= 46, *p *= .009; RMSEA = .038, 90%CI [.019, .054]; CFI = .983; AIC = 159.5). This model fit the data better. Because an independent Long-term Memory factor could not be identified, the indicators of Long-term Memory were deleted and the model re-estimated (χ^2 ^= 53.8, *df *= 29, *p *= .003; RMSEA = .047, 90%CI [.027, .066]; CFI = .979; AIC = 125.8); this model also fit the data well and to abbreviate the test battery the long-term memory indicators, that is, the delayed word recall and delayed face recognition tasks, were dropped from the test battery for future assessment sessions. This final model was taken as the measurement model for the accuracy-based tasks and is depicted in Figure 1.

**Figure 2:**
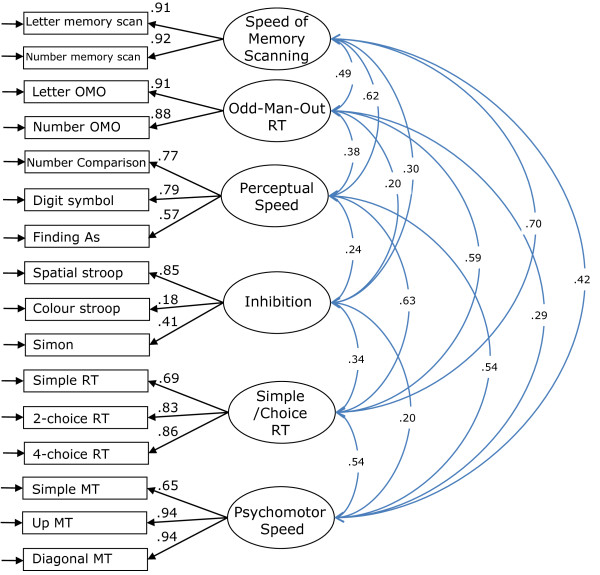
**Measurement Model for the tasks assessing speed of cognitive performance**. Final measurement model of the baseline data for speed tasks (χ^2 ^= 155.9, *df *= 89, *p *< .001; RMSEA = .044, 90%CI [.032, .055]; CFI = .979; AIC = 281.947). Correlations are represented by double-headed arrows. Abbreviations: RT = Reaction Time; MT = Movement Time.

For the speed-based tasks, the model specified latent variables for Simple/Choice Reaction Time, Odd-man-out Reaction Time, Inhibition, Speed of Memory Scanning, and Psychomotor Speed, with the indicators for each factor as specified under each construct in Appendix 1. The model fit was acceptable (χ^2 ^= 171.4, *df *= 104, *p *< .001; RMSEA = .041, 90%CI [.030, .051]; CFI = .979; AIC = 303.4) and, except for Inhibition, the indicator loadings were of moderate to high magnitude, in the expected direction, and significant; the loadings of the Inhibition tasks were generally lower but all were significant except Flanker, which was near zero (-.02). The magnitude of the factor correlations ranged from .19 to .70, all were in the direction indicating positive relationships, and none was so high as to imply redundancy of any factor. The Flanker task was deleted and the model re-estimated (χ^2 ^= 155.9, *df *= 89, *p *< .001; RMSEA = .044, 90%CI [.032, .055]; CFI = .979; AIC = 281.947); all coefficients were significant and the fit statistics for this model were similar but the AIC indicated better model fit. Although the loading of Colour Stroop on inhibition was also low (.18), given the more experimental nature of this factor (ie., not an established unitary construct in the literature) only the Flanker task was dropped from the test battery for future assessments. Figure 2 depicts the final speed measurement model.

In total, on the basis of the above analyses, three tasks from the initial test battery were dropped from the battery for the subsequent assessments.

## Conclusions

This methodology and protocol paper provides a detailed overview of the design and implementation of a double-blind, randomised, controlled trial assessing the efficacy of a DHA-rich fish-oil supplement on slowing cognitive decline, over 18-months, in cognitively-normal older adults. This is one of the few trials to date to examine the impact of *n*-3 LC PUFA supplementation on cognitive decline in cognitively healthy older adults. Advantages of the current study are the comprehensive assessment of cognitive functioning and the multiple measurement points, which allow for assessment of an intervention effect on a trajectory, rather than effects at a specific time point. The significance for society could be wide-ranging if such an available and inexpensive dietary treatment has the possibility to safely reduce cognitive decline in ageing, given the links between cognitive functioning and the societal and personal burden of dependency and decreased quality of life in older age.

## Abbreviations

AD: Alzheimer's disease; AIC: Akaike Information Criterion; ApoE: Apolipoprotein E; CES-D: Centre for Epidemiology Studies Depression Scale; CFA: confirmatory factor analysis; CFI: Comparative Fit Index; CFQ: Cognitive Failures Questionnaire; CRP: C-reactive protein; DHA: docosahexaenoic acid; EPA: eicosapentaenoic acid; FAME: fatty acid methyl esters; FFQ: food frequency questionnaire; HbA1c: glycated haemoglobin; HDL: high-density lipoprotein; HPLC: high performance liquid chromatography; LDL: low-density lipoprotein; LGCM: latent growth curve modelling; LLFDI: The Late Life Function and Disability Instrument; MDA: malondialdehyde; MMSE: Mini-mental State Examination; *n*-3 LC PUFA: omega-3 long-chain polyunsaturated fatty acid; PRMQ: Prospective and Retrospective Memory Questionnaire; PWI: the Personal Wellbeing Index; RCT: randomised controlled trial; RMSEA: Root Mean Square Error of Approximation; SF36V2: SF-36 version 2; SWLS: Diener's Satisfaction with Life Scale.

## Competing interests

The authors declare that they have no competing interests.

## Authors' contributions

VD conceived, designed, and implemented the study, designed and developed the cognitive test battery and designed the functional capacity/wellbeing assessment, drafted the manuscript, performed the statistical analyses, and interpreted the data. All authors contributed to development of the study design and protocol, were applicants for funding, reviewed the manuscript, and approved the final version.

## Appendix 1: Cognitive test battery

Parallel versions of each task were presented at each assessment visit unless otherwise specified. The Flanker task and the delayed recall tasks for Face Memory and Word Memory were only presented at the first assessment because these tasks were found not to adequately mark the expected latent construct in confirmatory factor analyses of the baseline results, conducted between assessments 1 and 2.

Tests were administered via paper-and-pencil or computer. The order of presentation of tests is indicated in parentheses following the name of the task below. All computerised task scripts, excepting Inspection Time, were written and/or modified using the software Inquisit (v. 2^©^, Millisecond Software). The Memory Scanning and Inhibition tasks were newly devised.

### Knowledge

Vocabulary (#17). 20 items from forms 1 and 2 of the standard, advanced, and extended range vocabulary tests from ETS Kit of Factor-Referenced Tests [71] comprise this multiple-choice task. A 4-minute time limit was imposed.

### Reasoning

Raven's (Standard Plus) Progressive Matrices (#10) [72]. Stimuli are a three by three array of symbols, with the bottom right hand symbol missing. Participants choose, from options given below the matrix, which symbol logically completes the matrix. Twenty items, selected to maintain progressive difficulty, were administered with a ten-minute time limit. The same version was administered at each assessment.

Letter Sets (#13). This task is from the ETS Kit of Factor-Referenced Tests [71]. Stimuli consist of a series of five, four-letter items, which follow a predetermined alphabetical ordering. The task is to determine the rule or pattern linking four of the items and cross-out the item not fitting the rule. Fifteen items comprised this test, administered with a seven minute time limit. The same version was administered at each assessment.

Everyday Problems Test (#19) [73]. This test is an objective measure of adults' ability to solve problems of daily living, presented as printed material, in seven domains considered critical for independent living. The seven domains, similar to those covered by the Instrumental Activities of Daily Living (IADL [74]), are health (medications), meal preparation/nutrition, phone usage, shopping, financial management, household management, and transportation. Stimulus material is in three formats - directions, charts, and forms - and number of questions is balanced across domains and format. For each item, a stimulus (e.g., prescription medication label, phone fee chart, mail-order form) is presented from which the participant must derive the answer through reasoning, to the accompanying item question. The test at each time point comprised 21 multiple-choice items (selected from the full-test 84-item pool), with a 15 min time limit imposed.

### Retrieval Fluency

Word Endings (#18). This test is derived from the ETS Kit of Factor-Referenced Tests [71]. The task is to write down as many words ending with a specific set of letters (e.g., 'in'). The score is the number of valid words written within 2 minutes.

Retrieval Fluency (#21). This test is derived from the Woodcock-Johnson III Tests of Cognitive Ability [75]. Participants have to write as many words falling within a given category as possible, in two minutes. The score is the number of things written, bar repeats.

### Short-term Memory

Face Memory - immediate recall (#1). This task, derived from Herzmann, Danthiir, Schacht, Sommer, and Wilhelm [76], is computerised. 20 photographic portraits are presented with 1.5 mins to study them. Each trial in the subsequent test period presents one of the studied faces next to a completely new face and the task is to indicate which face was the studied face.

First and Last Names (#11). In this paired associates test, derived from the ETS Kit of Factor-Referenced Tests [71], participants are given 2.5 mins to study a list of 15 first and last name pairs. They are then shown only the last names and allowed 1.5 mins to write down the associated first names.

Word Memory - immediate recall (#2). Word list A from the Rey Auditory Verbal Learning Test [77] was presented via computer at the first assessment and parallel word lists were presented for subsequent sessions. 15 words (nouns) were presented, one at a time, on screen for one sec duration, with an inter-trial interval of one sec. Immediately after presentation of the word-list, participants write down, in any order, as many of the words recalled.

### Long-term memory

Face Memory - delayed recall (#29). Approximately 3.5 hrs after presentation of the initial Face Memory task, participants were tested again on the faces studied in that task. Each trial presents one of the studied faces next to a completely new face. The task is to indicate the face that was studied in the initial study phase of the Face Memory task.

Word Memory - delayed recall (#6). Approximately 30 mins after presentation of the initial Word Memory task, participants write down as many words as they can recollect from the list, in any order.

### Perceptual Speed

Digit-Symbol Substitution (#12); from the revised version of the Wechsler Adult Intelligence Scale [78]. A code with nine symbols, each uniquely associated with one of the digits "1" through to "9", is presented at the top of the page, with rows of only the digits below. The task is to fill in beneath each digit, in order of presentation, as many of the corresponding symbols as possible, within 90 secs. The score is the number of correct answers.

Finding As (#9); from the ETS Kit of Factor-Referenced Tests [71]. Participants are required to examine columns of words; each column has five words containing the letter "A." The task is to place a line through words that contain "A" and ignore those that don't. The score is the number of words marked correctly within the one-minute time limit.

Number Comparison (#20); from the ETS Kit of Factor-Referenced Tests [71]. This task consists of two columns of number-pairs, the numbers varying from 3 to 12 digits in length. Participants are required to place a cross between pairs of numbers if they are not identical. The score is the number marked correctly within the one and a half minute time limit.

The following tasks are all computerised tasks.

### Working Memory

Each of the following tasks presents a processing task prior to the stimulus to be remembered. A reminder of the response keys for the processing task is present on the screen throughout. Each trial consists of two to five screens, depending on the difficulty level of the trial, prior to the cue to recall the stimuli. 12 items comprise the test, with three trials of set-size two, and two each of set-sizes three to five. The score is the mean of the proportion of numbers recalled in the correct serial position for each item.

Counting Span (#15) [79,80]. Participants must remember digits while concurrently undertaking a counting task. For each screen, a number of blue circles and squares, and green circles are shown. The participant must count and remember the number of blue circles in each screen, and then press a key indicating whether the number was odd or even. After a response, the next set of figures is presented and after the last set, participants have to serially recall the number of blue circles in each of the preceding displays, responding using the keyboard and mouse. If a participant does not correctly recall the serial position of a minimum of one digit in a set-size, the task ends.

Operation Span (#4) [79,80]. In this task, participants remember words (nouns) while concurrently solving a simple arithmetic problem. Each screen presents a maths equation followed by a word, of the form: "DOES (6 × 2) - 5 = 7? CLASS". Participants look at the word then press a key indicating whether the equation was correct or not. After the response, the next equation/word screen appears and after the final screen, participants write down the words recalled in the correct serial position. Parallel word sets were used in each session.

### Inspection Time

A windows based version of Inspection Time (#31) [81] was administered. Participants repeatedly view a target stimulus consisting of two vertical lines, joined at the top by a horizontal line, for varying durations. One vertical line is always shorter, with equiprobability. For each trial, the participant indicates by mouse-press whether the shorter line was on the left or right side. Using an adaptive staircase algorithm [82], a participant's inspection time is defined as the duration of stimulus exposure (in ms) which has an associated probability of 79% of making a correct response.

For the following reaction time tasks, unless otherwise specified, a practice session of 12 trials precedes the start of each test and each task consists of 30 trials. Feedback (accuracy) is presented after each practice trial. The stimuli in both the practice trials and the test proper are balanced such that an equal number of each potential response is required, and for tasks with more than one condition (e.g., Colour Stroop), task-relevant stimulus characteristics are also balanced across conditions.

### Simple and Choice-reaction Time

A simple (#3) and two- (#5) and four-choice (#8) reaction time tasks are included, modified from Danthiir, Wilhelm, and Roberts (Further evidence for a multifaceted model of mental speed: Factor structure and validity of computerised tasks, submitted). The stimuli in these tasks are digits, each associated with arrows pointing in different directions. In all tasks, the stimulus-response coding is present at the top of the screen. Participants must press the appropriate arrow response key, depending on which number is displayed. The number of possible responses increases from one in the simple reaction time task to four in the 4-choice reaction time task.

### Odd-man-out (OMO) Reaction Time

Two variants of the OMO task (Letter (#14) and Number (#23) OMO, modified from Danthiir et al. (Danthiir, Wilhelm, & Roberts: Further evidence for a multifaceted model of mental speed: Factor structure and validity of computerised tasks, submitted) are included. The OMO task requires an additional discrimination compared to typical CRT tasks. A string of eight equidistant stimuli are presented on screen. In each string, three stimuli are identical (ie., targets) and different from the other five. Two of the targets are always closer together in relation to the position of the third target. The task is to press a key to indicate if the odd-man-out (i.e., the target furthest from the two closer target stimuli) is to the left or right of the other targets.

### Speed of Memory Scanning

Two variants of Sternberg's memory scanning paradigm [83] were devised, measuring speed of scanning items in short-term memory; one task using numbers (#7) as stimuli and the other, letters (#16). A string of two to five stimuli are presented simultaneously on the screen for 2200 ms. A probe stimulus is then presented and remains onscreen until participants press a key to indicate whether the probe was in the preceding string.

### Inhibition (or Interference Control)

The following tasks that were devised tap inhibitory processes, also known as interference control or inhibition of prepotent responses. In all tasks the stimuli vary by a task-relevant and -irrelevant characteristic. The task-irrelevant characteristic is linked (e.g., spatially) via automatic prepotent response to the response dimension, providing two or three stimulus-response compatibility (SRC) conditions depending on the task; congruent, incongruent, and sometimes neutral. Trials are balanced in number across stimulus response congruency (SRC) condition and stimulus. During the test, if an incorrect response was given twice in a row for a particular trial-type (e.g., a green left oriented stimulus) a reminder of the correct response keys was presented for 5 sec.

Simon Task (#30). The typical Simon effect refers to the interference (manifesting both in delayed RT and increase in errors) experienced when the response required by a task is spatially opposite to the location of the stimulus, creating a stimulus-response conflict [84]. Stimuli in this task were a red or green circle, presented on the left or right side of the screen. Participants press the right SHIFT key if the stimulus is green and the left SHIFT key if it is red. Two conditions are present in this task; stimulus-response congruent, where the location of the circle is on the same side as the required response (e.g., a green circle on the right side of the screen) and incongruent, where the location of the circle is on the opposite side of the required response (e.g., a green circle on the left side of the screen). The task comprises 60 trials. The dependent variable in analyses is the difference in RT between the congruent and incongruent conditions.

Colour Stroop Task (#22). This task is based on the well-known Stroop effect [85]. Stimuli are the words blue, yellow, note, or engine, coloured yellow or blue, presented one at a time on the computer screen. The task is to press the appropriate key indicating the colour the word is displayed in. Three conditions are present in this task: stimulus-response congruent, where the word matches the colour it is presented in (e.g., the word "YELLOW, " coloured yellow); incongruent, where the word does not match the colour it is presented in (e.g., the word "YELLOW, " coloured blue); and neutral, where the word is not a colour word (e.g., the word "ENGINE, " coloured yellow). 90 trials are presented and the dependent variable in analyses is the difference in RT between the neutral and incongruent conditions.

Spatial Stroop Task (#25). This task is a measure of the so-called spatial Stroop effect (e.g., [86]). The word LEFT or RIGHT is presented on the left or right side of the screen. Participants press the Right SHIFT key for the word "RIGHT" and the Left SHIFT key for the word "LEFT." There are two conditions in this task; stimulus-response congruent, where the word matches the side it is presented on (e.g., the word LEFT presented on the left side of the screen) and incongruent, where the word does not match the side it is presented on (e.g., the word LEFT presented on the right side of the screen). 60 trials comprise this task and the dependent variable in analyses is the difference in RT between the congruent and incongruent conditions.

Flanker Task (#24). The flanker effect refers to the influence of flanking distracter stimuli on target identification, whereby identification is facilitated by response-congruent flankers and inhibited by response-incongruent flankers [87]. The target in this case was either the letter "S" or the letter "H, " presented in the centre of the screen, flanked simultaneously by two letters displayed either side of the target letter. If the target letter is an "H" participants press the left SHIFT key and if the target letter is an "S" participants press the right SHIFT key. There are three different conditions: the congruent condition where the flanking letters are all the same as the target letter, the neutral condition where the flanking letters (the letter "P") are different from either of the target letters, and the incongruent condition where the flanking letters are the alternative target letter (i.e. if the target letter is "H" the flanking letters are "S"s and vice-versa). 90 trials are presented. The dependent variable in analyses is the difference in RT between the neutral and incongruent conditions.

### Psychomotor Speed

For these tasks, participants use only the index finger of their preferred hand. For each task, ten practice trials and 20 test trials are given. The dependent variable is the time taken (in ms) between key presses. Following the prompt to begin, participants press either one key, repeatedly (Simple Movement Time task (#26)), or two keys (vertically adjacent - Up Movement Time task (#27); diagonally adjacent - Diagonal Movement Time task (#28)) as quickly as possible.

## Appendix 2: Functional Capacity, physical activity, and well-being questionnaires

Unless stated otherwise, if the original questionnaire specified the time-period to which the questions were directed, this was specified as one month in the current study; the exception was questionnaires relating to mood, which were maintained as pertaining to the previous week to assess state, as opposed to trait, affective states.

### Health status

The Australian SF-36 version 2 [55] (SF36V2) health survey standard form was used as a measure of health-related quality of life. The SF36V2 comprises 36 items from which are derived eight norm-based scale scores: Physical Function, Role Physical, Bodily Pain, General Health, Vitality, Social Function, Role Emotion, and Mental Health. Two summary scale scores are derived from sums of the eight scale scores, weighted by factor score coefficients, representing physical and mental health (physical component score (PCS) and mental component score (MCS)). The eight scale scores were normed according to 2004 data from a large South Australian population-based health survey [88,89] and the PCS and MCS were weighted using factor score coefficients computed in this population. On all scales, a higher score indicates better health.

The SF-36 was supplemented with the sleep scale from the Nottingham Health Profile [56], a generic validated health-related quality of life measure, to assess quality of sleep. The scale consists of five items weighted for severity, which have a maximum sum of 100. Participants answer yes or no to each item and the weights are added on items with a positive response; the higher the score the greater the health problem.

### Physical activity

The Yale Physical Activity Survey is a validated [57,90], interviewer-administered questionnaire specifically designed to be sensitive enough to capture the lower-intensity range of physical activities of older adults. It assesses the level of activity during a typical week in the last month. Minor changes to instructions were implemented to adapt the survey to a format suitable for self-completion. The questionnaire assesses work-, yardwork-, exercise-, caretaking-, and recreational-related physical activity, expressed as minutes per week, as well as current participation in several different types of activity categories believed to reflect physical activity dimensions. A total of eight indices are derived, three summary and five subscale indices, and a seasonal adjustment score can be calculated: 1) Total Time Summary Index, expressed as hours per week; 2) Energy Expenditure Summary Index, expressed as kcal/week^-1^; and 3) Activity Dimensions Summary Score (sum of five subscale indices, vigorous activity, leisurely walking, moving, standing, and sitting, each derived by multiplying frequency score by duration score for each activity type and then multiplied by a weighting factor (based on relative intensity of activity), expressed as a unit score), expressed as total units.

### Depression

Depression was measured by the Centre for Epidemiology Studies Depression Scale (CES-D) [49], a 20-item tool measuring depressive symptoms in the general population; it has been extensively validated, including on older adults [91-93] and is widely used as a screening measure. Frequency of occurrence of symptoms is reported for the past week (as in original) and a higher score is indicative of more depressive symptoms, with a maximum score of 60; scores of 16 to 26 are considered indicative of mild depression and scores of 27 or more indicative of major depression. Because the CES-D uses a 4-point response scale it is more likely to be sensitive to change than other commonly used measures with a 2-choice response format.

### Subjective well-being

#### Mood

The Positive and Negative Affect Schedule - extended version [52], is a widely validated 60-item scale that assesses two broad mood factors - positive and negative affect - as well as 11 specific affects. Participants indicated on a 5-point Likert scale the extent to which they had experienced the listed emotions over the last week, with higher scores indicating greater affect.

#### Life satisfaction

Two measures of life satisfaction were administered to participants; Diener's Satisfaction with Life Scale (SWLS) [53] and the Personal Wellbeing Index (PWI) [54]. Both of these measures were included to assess cognitive rather than affective assessments of subjective wellbeing.

The SWLS assesses respondents' global evaluation of life satisfaction by stating their level of agreement with five statements regarding their life, using a seven point Likert scale. The total score represents the extent of global life satisfaction, with higher scores indicating greater satisfaction and a maximum score of 35; normative data are available for diverse populations including older adults. The scale has been shown to be sensitive to possible changes in levels of life-satisfaction over time due to the occurrence of positive and negative life-experiences [94].

The PWI scale measures subjective wellbeing as an aspect of quality of life. The scale consists of eight items assessing satisfaction with various aspects of life and participants rate their level of satisfaction on an 11-point Likert Scale. These eight domain scores are summed to yield an average score which represents subjective wellbeing, higher scores indicative of greater satisfaction, with a maximum of 100.

### Self-reported cognitive functioning

Self-reported cognitive functioning in everyday situations was assessed by the Cognitive Failures Questionnaire (CFQ) [50] and the Prospective and Retrospective Memory Questionnaire (PRMQ) [51].

The CFQ comprises 25 questions measuring self-reported failures in everyday perception, memory, and motor function. Participants rated on a 5-point Likert scale the frequency of such events over the last 4 weeks. A total score is typically derived, measuring proneness to everyday slips and errors, with higher scores indicating greater perceived frequency of mistakes with a maximum score of 100. However, an investigation of the latent structure of the CFQ, using a large representative adult sample, suggests its items assess three constructs - Forgetfulness, Distractibility and False Triggering - which are sensitive to age-effects [95].

The PRMQ is a validated [51,96-98] self-report measure, comprising 16 questions, divided equally to assess the frequency of occurrence of prospective and retrospective memory errors in everyday life. Participants were asked to assess the frequency of these occurrences over the last 4 weeks, on a 5-point Likert scale. The PRMQ has been found to have a tripartite latent structure consisting of a general memory factor plus orthogonal specific factors of prospective and retrospective memory [98,99], thus the PRMQ total scale can be used as a measure of general self-rated memory or specific memory scores can be derived. The maximum total score is 80, with higher scores equating greater perceived frequency of memory mistakes.

### Functional Capacity

The Late Life Function and Disability Instrument [100,101] (LLFDI) was designed to assess meaningful change in functioning and disability in older community dwelling adults. Concurrent and predictive validity of the LLPDI has been assessed [102].

The Physical Function component of the LLFDI [101] comprises 32 items measuring the level of difficulty experienced in performing specific activities as part of daily routines. The activities are divided into three domains: basic lower extremity functioning, advanced lower extremity functioning, and upper extremity functioning, with additional questions for users of assistive devices. Respondents rate the difficulty experienced on a five-point Likert scale; all items can be added to derive a total functioning score, with a maximum of 160 and higher scores indicative of better physical functioning.

The Disability component of the LLFDI [100] comprises 16 items measuring two dimensions: the frequency of performance of specific activities as part of daily routines, and the extent of limitation experienced in performing the activities. Respondents rate the frequency of performance and the extent of limitation experienced on a five-point Likert-type scale. Two sub-domains are identified within each dimension: Frequency dimension - 1) Social role (performance of various social and community tasks) and 2) Personal role (performance of various personal tasks); Limitation dimension - 1) Instrumental role (activities at home and in the community) and 2) Management role (tasks that involve minimal mobility or physical activity). Distinct summary scores can be calculated to represent the Limitation and the Frequency dimension, each with a maximum score of 80 and higher scores indicating less limitation and greater frequency, respectively.

## Endnotes

^a ^The current trial commenced prior to completion of either of those trials.
